# Leukocyte Attraction by CCL20 and Its Receptor CCR6 in Humans and Mice with Pneumococcal Meningitis

**DOI:** 10.1371/journal.pone.0093057

**Published:** 2014-04-03

**Authors:** Matthias Klein, Matthijs C. Brouwer, Barbara Angele, Madelijn Geldhoff, Gabriel Marquez, Rosa Varona, Georg Häcker, Helga Schmetzer, Hans Häcker, Sven Hammerschmidt, Arie van der Ende, Hans-Walter Pfister, Diederik van de Beek, Uwe Koedel

**Affiliations:** 1 Department of Neurology, Ludwig-Maximilians-University, Munich, Germany; 2 Department of Neurology, University of Amsterdam, Amsterdam, The Netherlands; 3 Center of Infection and Immunity Amsterdam, Academic Medical Center, Amsterdam, The Netherlands; 4 Genetrix, Madrid, Spain; 5 Departamento de Immunologia y Oncologia, Centro National de Biotecnologia, Madrid, Spain; 6 Institute for Medical Microbiology and Hygiene, Albert-Ludwigs-University Freiburg, Freiburg, Germany; 7 Medical Department III, Ludwig-Maximilians-University, Munich, Germany; 8 Department of Infectious Diseases, St. Jude Children’s Research Hospital, Memphis, Tennessee, United States of America; 9 Department Genetics of Microorganisms, University of Greifswald, Greifswald, Germany; 10 Department of Medical Microbiology, University of Amsterdam, Amsterdam, The Netherlands; Washington University, United States of America

## Abstract

We previously identified CCL20 as an early chemokine in the cerebrospinal fluid (CSF) of patients with pneumococcal meningitis but its functional relevance was unknown. Here we studied the role of CCL20 and its receptor CCR6 in pneumococcal meningitis. In a prospective nationwide study, CCL20 levels were significantly elevated in the CSF of patients with pneumococcal meningitis and correlated with CSF leukocyte counts. CCR6-deficient mice with pneumococcal meningitis and WT mice with pneumococcal meningitis treated with anti-CCL20 antibodies both had reduced CSF white blood cell counts. The reduction in CSF pleocytosis was also accompanied by an increase in brain bacterial titers. Additional *in vitro* experiments showed direct chemoattractant activity of CCL20 for granulocytes. In summary, our results identify the CCL20-CCR6 axis as an essential component of the innate immune defense against pneumococcal meningitis, controlling granulocyte recruitment.

## Introduction

Bacterial meningitis accounts for 135,000 deaths in 1.2 million cases worldwide each year. Pneumococcal meningitis is the most common and most severe form of meningitis: 16–35% of the patients die and up to 50% of survivors suffer from long term sequelae [1], [2]. Animal experiments have elucidated the pathophysiology of pneumococcal meningitis. Physiologically low concentrations of leukocytes, antibodies, and complement components in the subarachnoid space allow fast proliferation of bacteria that have reached the subarachnoid space. Initially, the infection is sensed by pathogen recognition receptors including TLR2 and TLR4 [3], [4]. Subsequently, a complex network of cytokines and chemokines starts operating and leukocytes are attracted [5], [6]. Few of the cytokines and chemokines involved in this network have been assigned a function and a role.

Using protein array technology we showed that the chemokine CCL20 is up-regulated in the cerebrospinal fluid (CSF) of patients with pneumococcal meningitis [7]. CCL20 is also known as macrophage inflammatory protein 3α (MIP-3α), liver and action-related chemokine (LARC), and Exodus-1 [8]–[10] and is expressed in a broad spectrum of cell and tissue types [11]. In contrast to other known chemokines that usually bind to multiple chemokine receptors, CCL20 binds solely to the chemokine receptor CCR6, and CCL20 is the only known cytokine ligand for CCR6. This unique CCR6/CCL20 combination is involved in the chemoattraction of immature dendritic cells and effector/memory T- and B-cells in skin and mucosal surfaces [11]. In addition to their important role in autoinflammatory conditions such as inflammatory bowel disease [12], psoriasis [13] or autoimmune encephalitis (EAE) [14], CCL20 and CCR6 were detected in some bacterial infections, e.g., parodontitis [15] and *Helicobacter pylori* gastritis [16], [17], and they were shown to play a role in the generation and maintenance of the adaptive immune defense against bacteria in the gut [18]. In addition, CCR6 and CCL20 were found to be crucial for the inflammatory response in a model of peritonitis [19]. There have been no studies on the role of CCL20 and CCR6 in bacterial infections of the central nervous system.

Here, we assessed the role of CCL20 on cerebral inflammation by determining CCL20 levels in the CSF of patients with pneumococcal meningitis. As the infiltration of the subarachnoid space is dominated by neutrophils during pneumococcal meningitis, we next evaluated direct effects of CCL20 on neutrophil recruitment *in vitro* and *in vivo.* Finally, the findings were validated and explored in a well-established animal model of pneumococcal meningitis by blocking CCL20 pharmacologically and by the evaluation of CCR6-deficient mice.

## Materials and Methods

All clinical investigations were conducted according to the principles of the Declaration of Helsinki. Ethical approval was obtained from the Medical Ethical Committee of the Academic Medical Center, Amsterdam. Written informed consent was obtained from all participating patients or their legally authorized representatives. All animal experiments were approved by the Government of Upper Bavaria.

### Nationwide Prospective Community-acquired Bacterial Meningitis Cohort

From March 2006 to June 2010 patients older than 16 years of age with positive cerebrospinal fluid cultures who were identified by The Netherlands Reference Laboratory for Bacterial Meningitis were included in the study. Informed consent was obtained from all participating patients or their legally authorized representatives.

### CSF Analysis

CSF of pneumococcal meningitis patients was obtained from the diagnostic lumbar puncture. We selected 19 patients with normal CSF who underwent CSF examination to exclude subarachnoid hemorrhage and 24 patients with PCR proven viral meningitis as controls. CSF was spun down and supernatant was stored at −80°C until analysis. CSF CCL20 and IL-17 levels were determined using the luminex technology using a Milliplex assay (Millipore, Billerica, MA, USA) according to the manufacturer’s instructions.

### Experimental Pneumococcal Meningitis

A well-established mouse model of pneumococcal meningitis was used as previously described [5]. Briefly, mice were weighed and clinically examined by a clinical scoring system [5]. In healthy animals, the score was 0; infected animals that died within the observation period scored 16 points. Meningitis was introduced by transcutaneous injection of 15 μl of a bacterial suspension containing 10^7^ colony-forming units (cfu)/ml of *Streptococcus pneumoniae* type D39 *(S. pneumoniae, D39)* into the cisterna magna under short-term anesthesia with halothane. Then, animals were allowed to wake up. All animals that were studied for longer than 24 h after infection received antibiotic therapy with ceftriaxone 100 mg/kg qd, starting 24 h after infection. At the end of each experiment, the animals were weighed, scored clinically, and their temperature was taken. Then, the mice were anesthetized with 100 mg/kg ketamine and 5 mg/kg xylazine and a catheter was placed in the cisterna magna. Cerebrospinal fluid (CSF) samples were obtained for CSF leukocyte count (CSF–WBC). After deep anesthesia with ketamine, animals were killed using thiopental (300 mg/kg body weight, intraperitoneal application) and perfused transcardially with 15 ml ice cold potassium buffered saline (PBS) containing 10 U/ml heparin. The brain was removed and frozen immediately. For the assessment of survival, humane endpoints were used: animals that suffered from a clinical score >14 points, status epilepticus, body temperature <34°C or weight loss >20% were euthanized using thiopenthal (300 mg/kg body weight, intraperitoneal application). All animal experiments were carried out in strict accordance with the recommendations in the Guide for the Care and Use of Laboratory Animals (Institute of Laboratory Animal Resources, National Research Council, USA) and with the German Animal Protection Act. All animal studies were approved by the Committee on the Ethics of Animal Experiments of the Government of Upper Bavaria, Germany (Permit Numbers: 55.2-1-54-2531-32-04 and 55.2-1-54-2531-47-08).

#### Experimental groups

(i) For analysis of time-dependent CCL20 expression pattern, infected C57BL6 mice (Charles River) were studied at the following time points: 6 h (n = 6), 24 h (n = 9), 30 h (n = 6), 48 h (n = 6), 72 h (n = 6), and 120 h after infection (n = 7). (ii) To analyze the role of CCL20, C57BL/6 mice were infected and intraperitoneally treated with a monoclonal anti-CCL20 IgG antibody (R&D Systems, Cat.# MAB760) or IgG isotype control antibody (R&D Systems, Cat.# MAB005) 3 hours after infection (n = 10 per group). These mice were followed until 24 h after infection. (iii) For evaluation of the role of the receptor CCR6 on the pathogenesis of pneumococcal meningitis, *Ccr6* ^−/−^ mice (backcrossed into C57BL/6 background for >8 generations) [12], [20] were infected and compared with infected wild type (WT) control animals (C57BL/6). These animals were followed until 24 h (*Ccr6* ^−/−^ n = 12; WT n = 12) and 48 h (*Ccr6* ^−/−^ n = 12; WT n = 13) after infection. *Ccr6* ^−/−^ and WT mice were age- and sex-matched. (iv) The impact of CCL20 on the recruitment of leukocytes to the CSF in meningitis was evaluated by injection of recombinant CCL20 (R&D Systems, Cat.# 760-M3-025/CF, 20 ng/mouse) or heat-inactivated recombinant CCL20 into the cisterna magna of mice (n = 7) which were evaluated for CSF-WBC 8 h after injection. Differential CSF white cell counts were performed in 4 mice who received recombinant CCL20. In addition, we tested the CCL20 chemotactic capacity in combination with pneumococcal antigens by injection of heat-killed pneumococci and recombinant CCL20 (20 ng/mouse) or heat-inactivated recombinant CCL20 (10 minutes, 85°C) into the cisterna magna (n = 8) followed by determination of CSF-WBC 8 h after injection. (v) The possibility of a secondary IL-17 mediated pro-inflammatory effect of the CCL20/CCR6 axis was evaluated by treating mice with anti-IL17 IgG antibody (100 μg per mouse, R&D systems, Cat.# MAB421 [21]) or IgG2A isotype control antibody (100 μg per mouse, R&D systems, Cat.# MAB003) before infection; these mice were followed for 24 h and 48 h (with antibacterial treatment at 24 h after infection). For all experiments, animals that received an intracisternal injection of sterile PBS were used as controls (uninfected controls).

#### Cerebellar bacterial titers

The cerebellum was dissected and homogenized in 1 ml sterile PBS. Cerebellar homogenates were diluted serially, plated on blood agar plates, and cultured for 24 h at 37°C with 5% CO_2_.

#### Analysis of cerebral bleeding and hydrocephalus

Mice brains were cut in a frontal plane into 10 μm thick sections throughout the ventricle system and photographed (9 sections with 0.3 mm intervals). The brain and ventricle areas were measured (Image tool, UTHSCSA) and the volumes were estimated (∑ ventricle area/9 pictures×0.3 mm). Haemorrhagic spots were counted and the bleeding area was measured.

#### ELISA

Albumin content in brain homogenates was measured by ELISA to assess blood-brain barrier integrity as previously described [22]. Protein levels in mice brains for CCL20 (R&D Systems, Cat.# MCC200), IL-1β, IL-6, and IL-17 as well as IL-22 in the CSF of mice were assessed by ELISA according to the manufacturer’s instructions.

### Immunohistochemistry

Paraffin-embedded brain tissue of infected and healthy control mice was deparaffinized and boiled in 10 mM citrate buffer (pH 6) in a microwave for antigen retrieval. Endogenous peroxidases were blocked by incubation with 0.3% hydrogen peroxide in methanol (MERCK, Hohenbrunn, Germany). Sections were incubated with anti-CCL20 antibody (Abcam, Cambridge, UK, ab9829) in blocking solution or (vi) with blocking solution without primary antibody (negative control). Bound primary antibody was detected using biotinylated goat anti-rabbit IgG (Vector Labs, Burlingame, CA, USA) as well as streptavidin horseradish peroxidase (DAKO, Hamburg, Germany) and diaminobenzidine (Vector Labs, Burlingame, CA, USA), which yields a brown reaction product. Counterstaining was performed using Mayer’s hematoxylin.

### Cell Culture

Hoxb8-immortalized neutrophil progenitors derived from C57BL/6 mice [23], [24] were cultured in Optimem medium (Invitrogen) supplemented with 10% FCS, 30 mM *β*-mercaptoethanol (Sigma), P/S, 4% supernatant (SN) from stem cell factor (SCF)-producing Chinese Hamster Ovarian cells, and 1 mM oestrogen (Sigma). Neutrophil differentiation was induced by removal of oestrogen, and subsequent culture for 4 days in medium containing 2% SCF SN.

### Flow Cytometric Analysis

Hoxb8 neutrophil suspensions with a cell concentration of 4×10^6^ cells/ml were incubated either with medium or with 10 ng/ml TNF-α for 20 min, as described in a recent study [25]. Then, cells were labeled with rat monoclonal antibodies directed either against Gr-1 (conjugated with FITC), Mac-1 (conjugated with PC7; both from eBioscience, San Diego, USA) or CCR6 (Rat anti-mouse CCR6 IgG2a, conjugated with PE; from R&D Systems) according to the manufacturer’s instructions. Appropriate isotype antibodies served as controls. The samples were analyzed using an FACS scan and Cell Quest Pro Software (Becton Dickinson). Neutrophils were gated using their characteristics in side and forward scatter. Their identity was further confirmed by their GR-1 and Mac-1 signals. Results are expressed as per cent positive cells in the appropriate gate. The markers were set according to the IgG isotype controls. In selected experiments, bone marrow-derived neutrophils were used instead of HoxB8 neutrophils. BMN were isolated as described previously with minor modifications [26]. Briefly, bone marrow cells were flushed from femurs with ice cold PBS containing 10% FCS. Neutrophils were then isolated from the cell suspension by density gradient centrifugation on Percoll (at 1,000 g for 30 min at 4°C). The Percoll density gradient was prepared in a 15 ml tube by layering 4 ml of 57% Percoll solution on top of 4 ml of 80% Percoll solution. The cell band that formed between the 81% and 57% layer was harvested. Cells were diluted in Opti-Mem medium containing 0.5% Nutridoma-SP (2×10^6^ cells/ml). The number and purity of BMN were analyzed by a conventional smear with Diffquick staining. BMN were then used for FACS analysis and chemotaxis experiments as described.

### Chemotaxis Assay

Neutrophil migration was assayed using a 48-well microchemotaxis chamber (Neuroprobe, Bethesda, MD) [27]. Briefly, CCL20, diluted in RPMI 1640, was placed in the lower well (25 μl). HoxB8 neutrophils (2×10^6^ cells/ml) were added to the upper well, which was separated from the lower well by a micropore filter (pore sizes 5 μm). After 120 min, cells were fixed with methanol and stained with DiffQuik (Baxter Diagnostics AG). Chemotaxis was quantified by microscopic counting of cells that migrated completely through the filter pores in 10 randomly chosen high-power fields. Cell migration was expressed as the mean number of leukocytes that migrated per field. FMLP was used as a positive control at concentrations of 10^−6^ M found to be optimal for the migration of neutrophils.

### Statistical Analysis

SYSTAT 9 (SPSS, Illinois, USA) was used for statistical analysis. Student’s t-test was used for comparison of two independent experimental groups at a certain time point unpaired. Differences were considered significant at p<0.05. The Mann-Whitney test with α-correction was used for evaluation of CCL20 expression at different time points. Data are expressed as mean ± standard deviation.

## Results

### CCL20 during Acute Pneumococcal Meningitis in Humans

**Figure 1:**
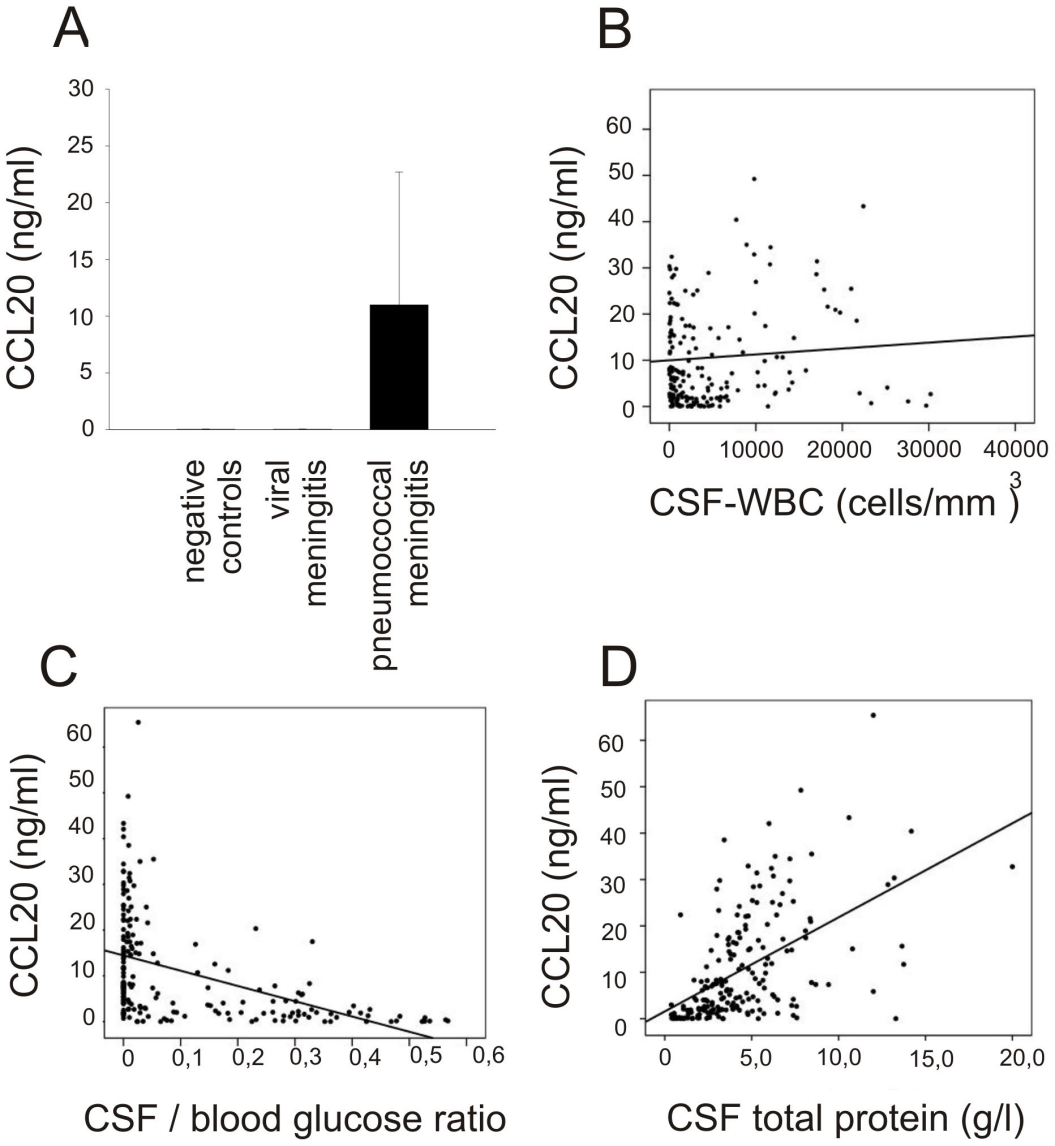
In patients with acute bacterial meningitis, (A) CCL20 cerebrospinal fluid (CSF) levels were significantly elevated compared with controls. CCL20 CSF levels of patients with pneumococcal meningitis correlated to (B) CSF white blood cell (WBC) counts, (C) CSF blood glucose ratio, and (D) CSF protein levels (see text for details).

To evaluate CCL20 during acute pneumococcal meningitis in humans we used patients from a nationwide prospective cohort study, performed from 2006–2010. In this study, 526 patients (73%) suffered from *S. pneumoniae* meningitis, *Neisseria meningitidis* was found in 90 (13%) episodes, *Listeria monocytogenes* in 35 (5%) episodes, and others in 68 (9%) episodes [28]. CSF from diagnostic lumbar puncture was available for 203 of 526 episodes of pneumococcal meningitis (39%). We also selected 19 patients with normal CSF who underwent CSF examination to exclude subarachnoid hemorrhage and 24 patients with viral meningitis as controls. In patients with pneumococcal meningitis, the median CSF CCL20 concentration was 6.9 ng/ml (interquartile range [IQR] 1.20–17.1). 96% of samples were above the lower limit of detection (33 pg/ml). CCL20 levels in negative controls and patients with viral meningitis were significantly lower than those of bacterial meningitis patients (Figure 1A). CCL20 CSF levels were correlated to CSF white blood cell (WBC) counts (Figure 1B, Coefficient: 0.175, p = 0.019), CSF blood-glucose ratio (Figure 1C, Coefficient: −0.388, p<0.001), and CSF protein levels (Figure 1D, Coefficient: 0.576, p<0.001).

### Expression Profile of CCL20 in a Mouse Model of Pneumococcal Meningitis

**Figure 2:**
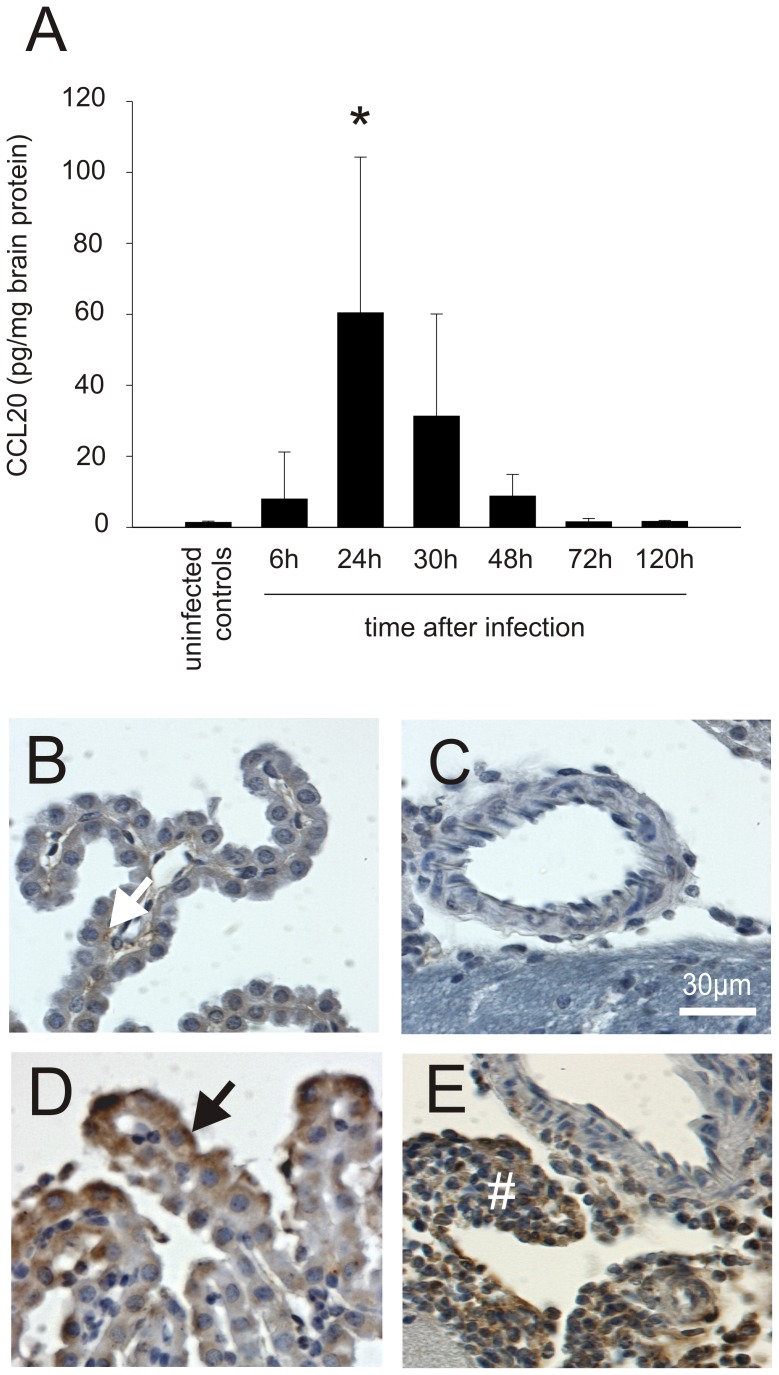
CCL20 is expressed mainly during acute pneumococcal meningitis. (A) Increased CCL20 levels were found in mice brain homogenates during acute bacterial meningitis using ELISA. After initiation of antibiotic therapy (starting 24 h after infection), they decreased quickly to normal ((*) p<0.01 compared with uninfected controls). In uninfected control mice, (B, C) only a very subtle CCL20-positive staining was observed (white arrow). In contrast, in animals with pneumococcal meningitis, CCL20-positive staining was found in (D) epithel cells of the choroid plexus (black arrow) and (E) the subarachnoid inflammatory infiltrate (#). Number of animals: 6 h: n = 6, 24 h: n = 9, 30 h: n = 6, 48 h: n = 6, 72 h: n = 6, and 120 h: n = 7. Uninfected animals were used as controls (n = 11).

To confirm that CCL20 is expressed in the meningitis mouse model, we examined mouse brain homogenates from WT mice infected with *S. pneumoniae*. At 6 and 24 hours after infection, CCL20 levels were increased (Figure 2A). At time points later than 48 h after infection, CCL20 levels had returned to normal. Low concentrations of CCL20 were detected in uninfected animals (Figure 2A). Immunohistochemical staining was positive for CCL20 in the leukocyte infiltrate in the subarachnoid space and in the plexus epithelium in mice subjected to pneumococcal meningitis (Figures 2B–E). CCL20 staining was undetectable in parenchymal cells (like migroglia) and within vessel walls.

### CCL20 Blockage and CCR6 Deficiency Lead to a Diminished Cerebral Immune Response and Poor Outcome in Experimental Pneumococcal Meningitis

**Figure 3:**
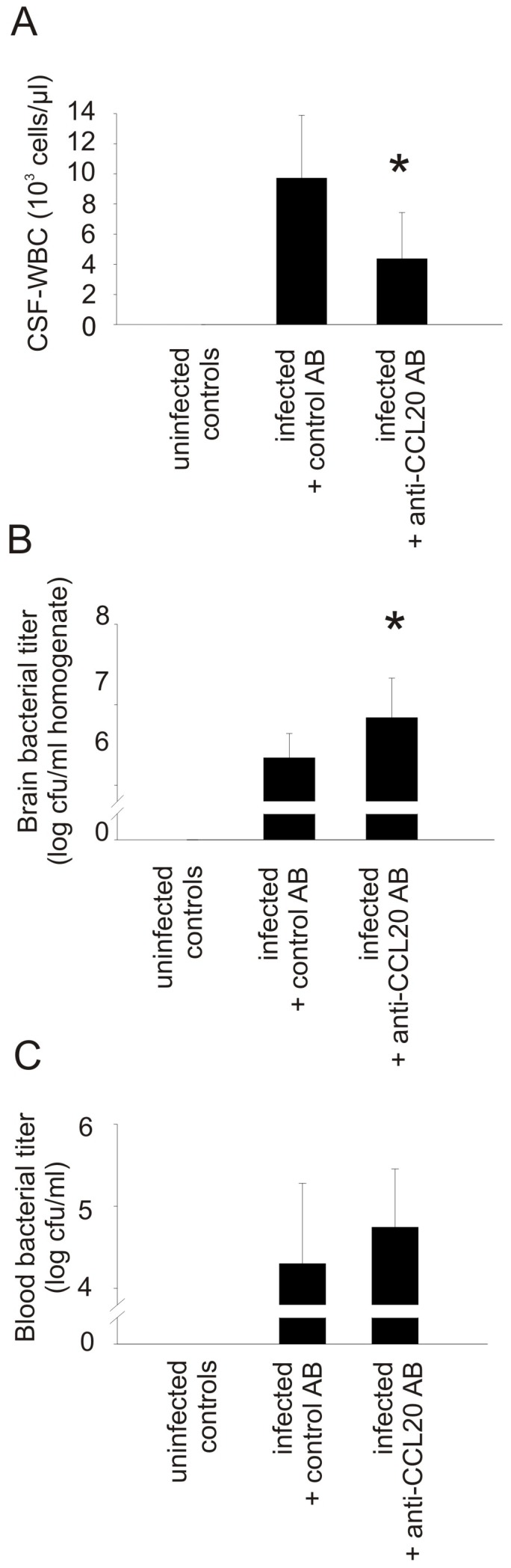
Anti-CCL20 antibody therapy lowers CSF inflammation *in vivo.* Although blockage of CCL20 using an anti-CCL20 antibody was not reflected in clinical differences, it resulted (A) in a decrease of CSF pleocytosis 24 h after infection. (B) This was associated with increased brain bacterial titers. (C) Blood bacterial titers were not affected. (*) p<0.05 compared with infected animals that received control antibodies. AB = antibody. N = 10 per group.

Then we aimed to decipher the functional role of CCL20 in our well-established mouse model using anti-CCL20 antibodies and CCR6-deficient mice. Treatment with anti-CCL20 antibodies resulted in lower CSF-WBC counts (Figure 3A) and impaired killing of bacteria compared to mice receiving isotype control antibodies (Figure 3B). Clinical scores, brain volumes, and the number of cerebral hemorrhages were similar in the investigated groups (data not shown). Furthermore, no differences were observed in blood-brain barrier damage, as well as brain IL-1β, MIP-2 or IL-6 levels (data not shown).

**Figure 4:**
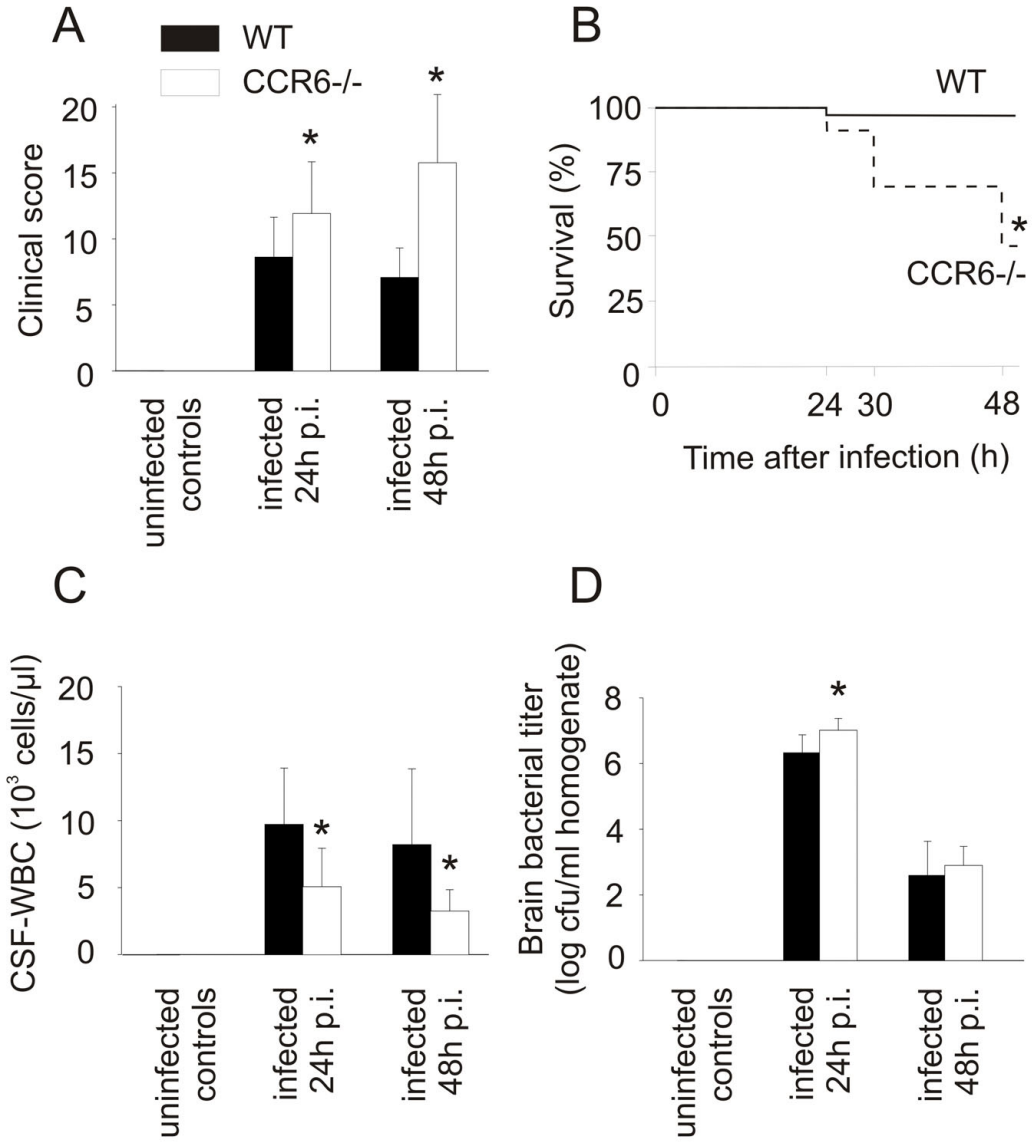
CCR6-deficient mice are more susceptible to pneumococcal meningitis. CCR6-deficient mice were more strongly affected from a clinical perspective by infection with *Streptococcus pneumoniae* than wild type controls. This was reflected (A) in increased clinical scores and (B) mortality. (C) CSF pleocytosis was lower in infected CCR6-deficient animals 24 h and 48 h after infection. (D) This was associated with higher brain bacterial titers at the time of initiation of antibiotic therapy (24 h after infection). (*) p<0.05 compared with infected wild type animals. Number of animals: 24 h: *Ccr6* ^−/−^ n = 12, WT n = 12; 48 h: *Ccr6* ^−/−^ n = 12, WT n = 13.

**Figure 5:**
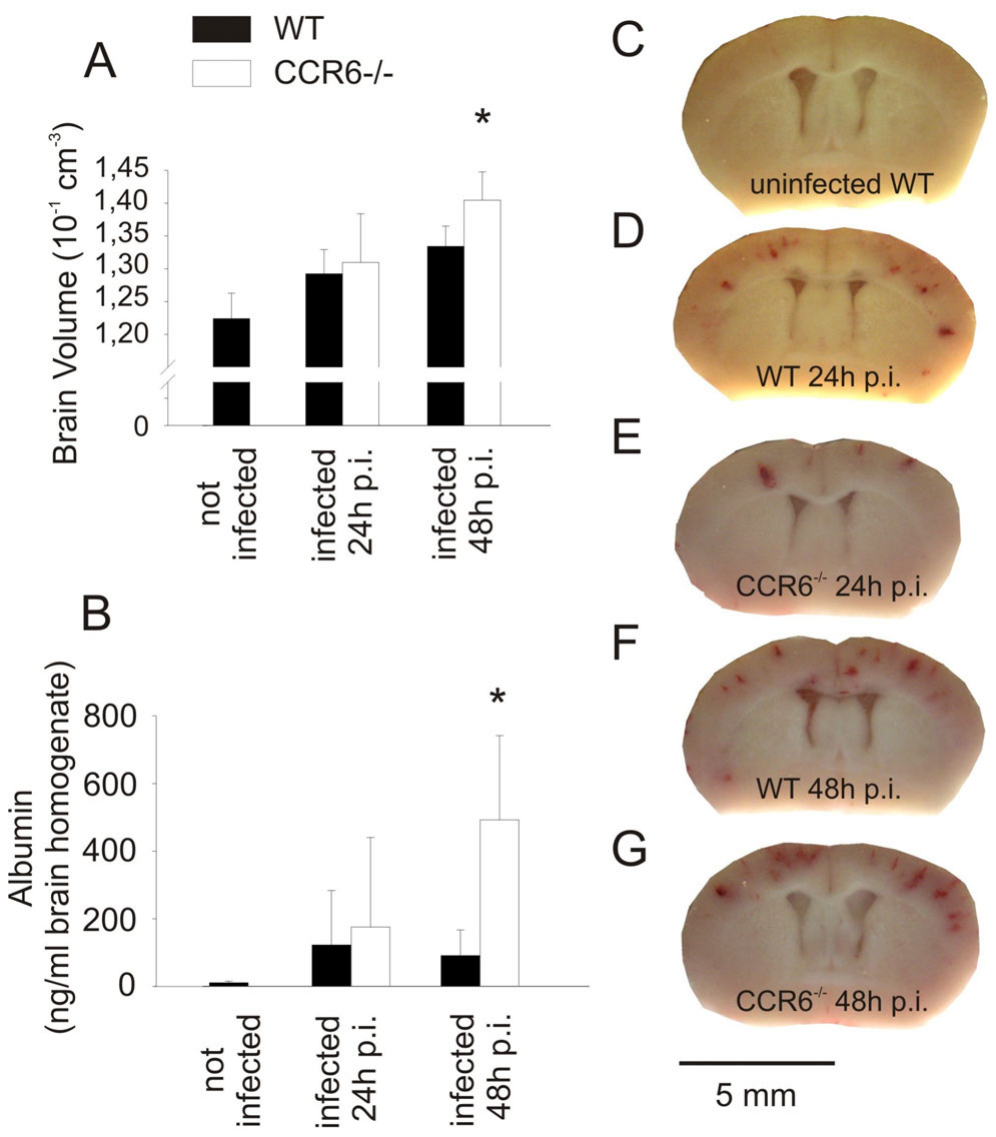
CCR6-deficient mice suffer from increased brain edema after antibiotic therapy. (A) Infected mice with CCR6-deficiency developed more pronounced brain edema than infected wild type mice 48 h after infection, reflected in an increase in the estimated brain volume. (B) At the same time point, an increase of brain albumin content was noted in infected *Ccr6* ^−/−^ mice, indicating blood-brain barrier disruption. These differences were only seen after but not before initiation of antibiotic therapy (24 h after infection). (C–G) Intracranial bleeding was similar in infected *Ccr6* ^−/−^ and wild type mice. (*) p<0.05 compared with WT control animals. Number of animals: 24 h: *Ccr6* ^−/−^ n = 12, WT n = 12; 48 h: *Ccr6* ^−/−^ n = 12, WT n = 13.

CCR6 is the only currently known receptor for CCL20. *Ccr6* ^−/−^ mice developed more severe disease than WT control animals reflected by higher clinical scores 24 h after infection (Figure 4A) and increased mortality 48 h after infection (Figure 4B). 24 h after infection, *Ccr6^−/−^* mice had lower CSF-WBC counts (Figure 4C,) and increased bacterial titers in the brain (Figure 4D). Brain IL-6, IL-1β, and MIP-2 levels of *Ccr6* ^−/−^ mice and WT mice were similar at 24 h (data not shown). At 48 h after infection, *Ccr6* ^−/−^ mice had lower CSF-WBC counts (Figure 4C), but increased brain volumes (Figure 5A) and an increased blood-brain barrier breakdown (Figure 5B) compared to infected WT mice. At 48 h after infection, bacterial titers (Figure 4D) and the number of cerebral bleedings in *Ccr6* ^−/−^ and WT mice were similar (Figure 5 C–F). In summary, *Ccr6^−/−^* mice suffering from meningitis were affected more strongly than wild type mice, as evidenced by an increase in clinical score and increased mortality.

### The CCL20-CCR6 Axis has a Chemoattractant Function

**Figure 6:**
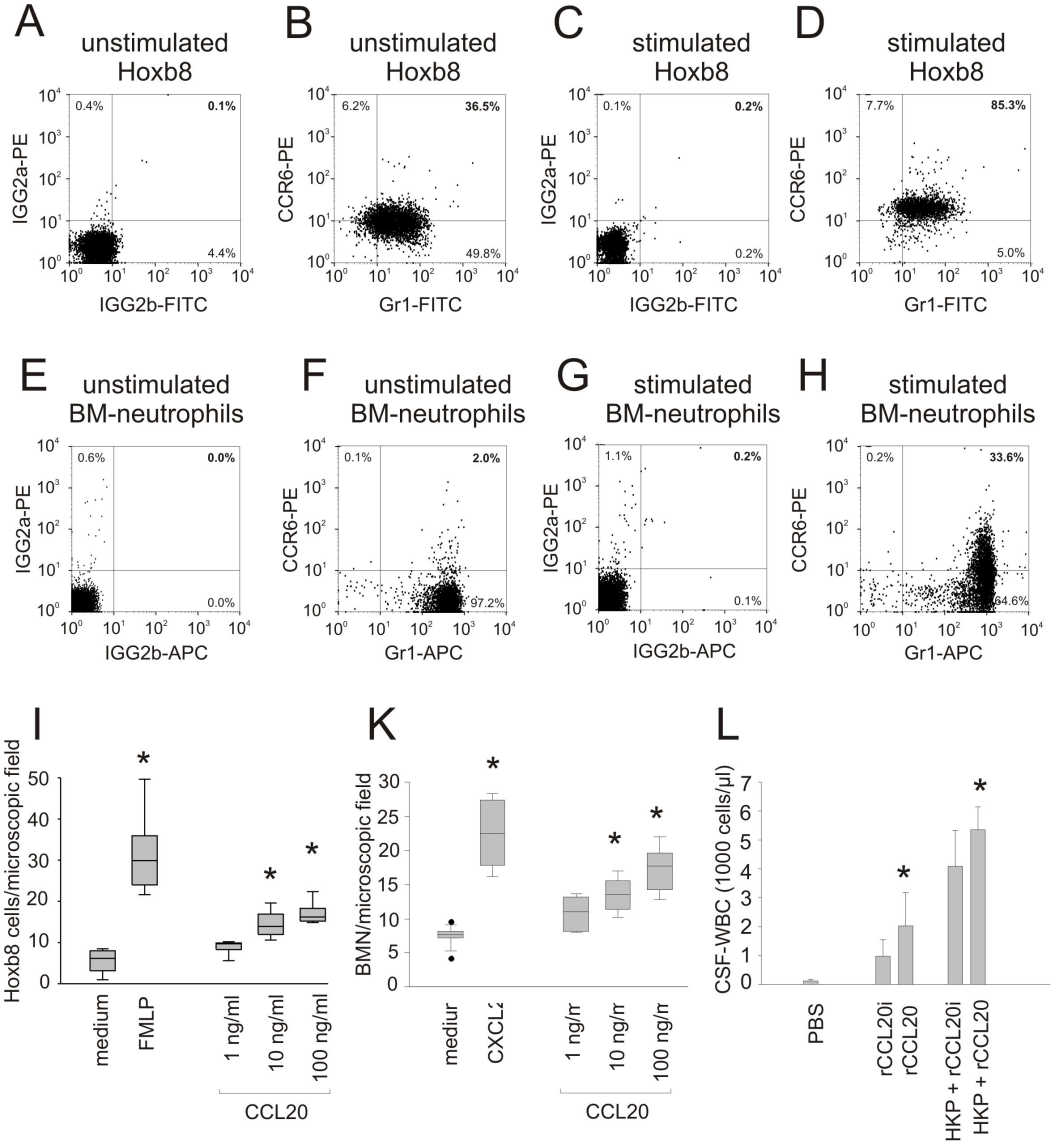
CCL20 chemoattracts granulocytes *in vitro* and *in vivo.* HoxB8 precursor cells could be differentiated into neutrophils, as indicated by the expression of Gr1. (A, B) The differentiated neutrophils were CCR6 positive. (C, D) Stimulation with TNF-α led to a marked increase of CCR6 expression in differentiated Hoxb8 neutrophils. Whereas (E, F) unstimulated bone marrow-derived neutrophils were CCR6-negative, (G, H) CCR6 became detectable on these cells upon exposure to heat-killed pneumococci (HKP). Using a chemotaxis assay, (I) differentiated Hoxb8 neutrophils and (K) bone marrow derived neutrophils migrated towards CCL20 in a dose-dependent way, demonstrating an *in vitro* chemotactic effect of CCL20 protein on CCR6 positive granulocytes. FMLP and CXCL2 were used as positive controls. (*) p<0.05 compared with Hoxb8 exposed to medium. (H) After intrathecal injection of recombinant CCL20 (rCCL20, n = 7) in wild type mice, a chemotactic effect was seen in comparison to injection with heat inactivated rCCL20 (rCCL20i, control, n = 7). In addition, rCCL20 co-administration to HKP led to a significant increase of the CSF-pleocytosis compared with rCCL20i co-administration to HKP (n = 8 for each group). (*) p<0.05 compared with rCCL20i or HKP+rCCL20i respectively.

To test a direct function of CCR6/CCL20 on mouse neutrophils, we used an *in vitro* system where neutrophils can be differentiated from HoxB8-transformed progenitors [23]. *In vitro*, undifferentiated precursor cells (data not shown) and differentiated (Gr1-positive) neutrophils were found to express CCR6 (Figure 6A and B). The CCR6 expression on differentiated Gr1-positive neutrophils increased after stimulation with TNF-α (Figure 6C and D). Whereas unstimulated neutrophils derived from the bone marrow of C57BL/6 mice were CCR6-negative (Figure 6E and F), CCR6 was found on these cells upon exposure to HKP (Figure 6G and H). This expression increased in a time-dependent fashion (data not shown). Using a chemotaxis assay, we found that both HoxB8 neutrophils and bone marrow-derived neutrophils migrated towards CCL20 in a dose-dependent manner (Figure 6I and K). *In vivo*, recombinant CCL20 amplified the CSF pleocytosis elicited by heat-killed pneumococci 8 h after infection (Figure 6I). Also, intracisternally applied CCL20 alone (without heat-killed pneumococci) had a mild chemotactic activity because CSF pleocytosis was significantly increased as compared with animals that received an intracisternal injection of heat inactivated CCL20 (Figure 6L). CCL20 mainly attracted granulocytes as differential CSF white cell counts revealed 72±13% granulocytes, 19±9% lymphocytes, and 9±3% monocytes.

### The Chemoattractant Activity of CCL20 is Independent of IL-17A/F and IL-22

**Figure 7:**
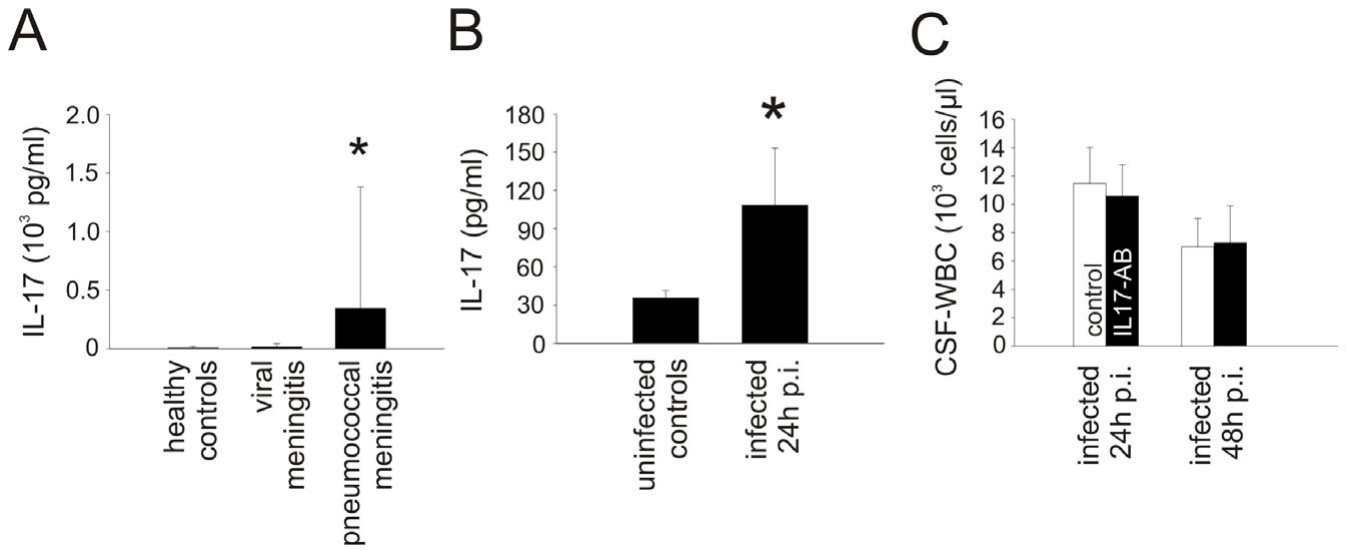
The pro-inflammatory effect of the CCL20/CCR6 axis seems independent of IL-17 production. IL17 was up-regulated in the CSF of (A) humans and (B) mice with pneumococcal meningitis. (C) However, antibody blockage of IL-17 in experimental pneumococcal meningitis did not lead to a reduction of inflammation in the CSF. P<0.05 as compared with uninfected controls. Number of animals: n = 5 mice per group.

Several reports have suggested that CCL20 leads to recruitment of CCR6-positive Th17 cells [29]. Th17 cells are potentially important for the initiation of early inflammation by the production of IL-17 or IL-22 [30]. To evaluate the influence of IL-17 in patients with pneumococcal meningitis we measured CSF levels of IL-17 in 203 patients and 42 controls. IL-17 was up-regulated in the CSF of patients with bacterial meningitis compared to controls (Figure 7A), but CSF IL-17 levels did not correlate with CSF-WBC counts (r = −0.049, p = 0.513). In WT mice with pneumococcal meningitis, IL-17 expression was higher than in uninfected wild type controls (Figure 7B). However, blockage of the heterodimer IL-17A/F by antibody treatment did not reduce inflammation 24 h and 48 h after infection (Figure 7C). Thus, IL-17A/F seems unlikely to be involved in the chemoattractant activity of the CCL20/CCR6 axis during pneumococcal meningitis.

Furthermore, we determined IL-22 concentrations in murine brains in order to gain insight into its possible involvement in the biological activity of the CCL20/CCR6 axis. IL-22 was elevated in brains of mice with pneumococcal meningitis as compared with uninfected controls at 24 h after infection (uninfected controls vs. infected wild type mice: 1.82±0.29 pg/mg brain protein vs. 4.08±1.51 pg/mg brain protein, p<0.001) and 48 h after infection (uninfected controls vs. infected wild type mice: 1.37±0.19 pg/mg brain protein vs. 3.15±1.78 pg/mg brain protein, p = 0.012). However, we did not detect any differences in IL22 levels between infected WT and CCR6^−/−^ mice at 24 h after infection (infected wild type mice vs. infected CCR6^−/−^ mice: 4.08±1.51 pg/mg brain protein vs. 3.39±1.84 pg/mg brain protein, p = 0.37) or at 48 h after infection (infected wild type mice vs. infected CCR6^−/−^ mice: 3.15±1.78 pg/mg brain protein vs. 2.59±0.59 pg/mg brain protein, p = 0.74). This observation argues against an essential role of IL-22 as a downstream mediator of CCL20/CCR6-induced pleocytosis.

## Discussion

Our results identify the CCL20-CCR6 axis as an important component of the innate immune defense against pneumococcal meningitis, controlling leukocyte recruitment and leukocyte-mediated bacterial killing in an IL17-independent manner.

The hallmark of pneumococcal meningitis is severe inflammation of the meninges and the subarachnoid space [31]. The inflammatory response is initiated and maintained by the expression of multiple cytokines such as Il-1β, KC, and MIP-2 [5]. High levels of CCL20 in the CSF of humans have been detected by protein array technology in patients with acute pneumococcal meningitis but not in control patients or in patients after recovery [7]. Here, the semi-quantitative protein array data was confirmed by quantitative analysis using a Luminex assay, which showed markedly enhanced levels of CCL20 in the CSF of pneumococcal meningitis patients included in a large prospective cohort study [32].

Time course analysis in a well-established mouse model of pneumococcal meningitis demonstrated that CCL20 was exclusively up-regulated during the acute stage and levels returned to normal quickly after administration of antibiotic therapy. CCL20 has recently also been shown to be produced in response to other bacterial infections. For example, CCL20 was detected in the gastric tissue of patients with *H. pylori* infection, and challenge of gastric epithelial cell lines with *H. pylori* induced the expression of CCL20 [16], [33]. This expression was triggered by the activation of NF-kappa B. Furthermore, *in vitro* CCL20 production was found in response to Pam3CSK4, interacting with TLR2, and LPS, interacting with TLR4 [34], [35]. Previously, we have demonstrated that pneumococcal challenge of the central nervous system activates the innate immune system via TLR2 and TLR4 and leads to an activation of NF-kappa B [4]. Thus, it appears possible that expression of CCL20 is mediated by TLR2 and TLR4 activation during pneumococcal meningitis.

Using a mouse model of pneumococcal meningitis, we could demonstrate a significant function of CCL20 and CCR6 in pneumococcal meningitis: whenever the CCL20-CCR6 axis was disabled, either by blockage of CCL20 using an anti-CCL20 antibody or by using CCR6-deficient mice, we noted a significant reduction of the CSF pleocytosis. This suggested a role of CCL20 in the recruitment of leukocytes in response to pneumococci. Chemotactic properties of CCL20 have previously been observed *in vitro* for mononuclear cells, T-cells [17], [36], and dendritic cells [37]. Also, neutrophils isolated from humans have been shown to express CCR6 and migrate towards CCL20 [38]. *In vivo,* a chemotactic effect of the CCL20/CCR6 axis has been demonstrated in diseases such as rheumatoid arthritis and autoimmune encephalitis. *In vivo* studies on bacterial infections have, so far, been limited to a description of an increase of CCL20 and CCR6-positive cells at the site of infection [15]–[17], [33], [39]. A significant functional role of CCR6 has only been shown for macrophage attraction and subsequent T-cell activation in response to skin infection of mice with *Salmonella species* [40]. Furthermore, the immune response was diminished in mice lacking CCR6 when multimicrobial peritonitis was induced by cecal ligation and puncture [19]. Further functional data on the recruitment of neutrophils by CCL20 and CCR6 during acute bacterial infections are sparse. Here, we first confirmed *in vitro* that neutrophils (MAC-1 positive cells differentiated from progenitors) express CCR6 and migrate towards CCL20, suggesting a direct chemotactic effect of CCL20 in neutrophil recruitment. Then, we demonstrated that, in mice, intercisternal administration of CCL20 by itself results in leukocyte recruitment into the CSF and also enhances CSF pleocytosis elicited by intrathecal injection of pneumococcal components (heat-inactivated pneumococci). Combined with the observation that blockage of CCL20 and deficiency of CCR6 reduced CSF pleocytosis after intracisternal pneumococcal challenge, our data indicate a crucial role of CCL20 in the recruitment of granulocytes to the subarachnoid space during pneumococcal meningitis. Since [i] CCL20 attracts granulocytes *in a Boyden chamber assay* and [ii] intracisternal CCL20 injection leads to CSF pleocytosis with granulocytes as the predominant cell type, we postulate a direct chemotactic role of CCL20 in the recruitment of granulocytes in pneumococcal meningitis. However, CCL20 might also contribute to granulocyte invasion into the CSF indirectly by attracting and/or activating other cell types that in turn promote granulocyte immigration into the subarachnoid space.

Several reports have suggested that CCL20 leads to recruitment of CCR6-positive Th17 cells. Th17 cells are the main producers of IL-17 which in turn has been found to be elevated in children with bacterial meningitis and a pro-inflammatory role was postulated [41]. Here, we could show an elevation of IL-17 in pneumococcal meningitis in adults, but we did not find a correlation of IL-17 levels and CSF-WBC. Furthermore, blockage of IL-17 in the experimental meningitis model did not diminish the immune response. Thus, IL-17 does not seem to mediate the pro-inflammatory effect of the CCL20/CCR6 axis in pneumococcal meningitis. Also, Th17 cells are known to produce IL-22. However, elevated IL-22 levels were not altered in CCR6-deficient mice in comparison to infected wild type mice. This missing difference in brain IL-22 levels argues against a critical role of this TH17-cytokine as a downstream mediator of CCL20/CCR6-induced leukocyte recruitment to the CSF in pneumococcal meningitis.

So far, CCL20 is the only known chemokine ligand of CCR6 [42]. Blocking CCL20 by antibodies was less effective than using CCR6-deficient animals in terms of the clinical course of the disease. Most likely, this might be explained by a generally less powerful impact of using functionally blocking antibodies *in vivo* in comparison with using genetically modified animals. Alternatively, other, as yet undetermined chemokine ligands or non-chemokine ligands of CCR6 like some β-defensins [43] could contribute to CCR6-mediated effects in meningitis and explain this discrepancy.

At the time when antibiotic therapy was started, we found significantly higher bacterial loads in infected *Ccr6* ^−/−^ mice than in infected WT mice. This phenomenon can be explained by decreased bacterial killing due to insufficient attraction of granulocytes. Similarly, infected mice lacking MyD88 or TLR2 and TLR4 (who also have an impaired immune response that manifests in a diminished CSF pleocytosis) are known to suffer from higher bacterial brain titers [4], [22]. Another explanation for the increase of bacterial titers after capture of CCL20 could be the previously described antibacterial property of CCL20 [44] but, *in vitro*, incubation of D39 with CCL20 did not reduce pneumococcal growth (data not shown). Thus, an antibacterial effect of CCL20 on *S. pneumoniae* does not seem to play a role in pneumococcal meningitis.

The higher numbers of *S. pneumoniae* D39 in *Ccr6* ^−/−^ mice might be the reason for the more pronounced clinical deterioration after antibiotic therapy. *S. pneumoniae* D39 is a potent inducer of cell death, pneumolysin being identified as one pneumococcal toxin that mediates such damage. For example, previous reports demonstrated reduced endothelial damage upon exposure to pneumolysin-deficient bacteria, as compared to the wild type strain [45], [46]. *In vivo*, higher bacterial titers lead to higher levels of pneumolysin, which is released especially after initiation of pneumococcal killing by antibiotic therapy [47]. Here, clinical deterioration after antibiotic therapy was associated with an elevation of blood-brain barrier breaching and brain edema. In consequence, the poor outcome in infected *Ccr6* ^−/−^ mice after antibiotic therapy can most likely be attributed to the increase of bacterial outgrowth, resulting in higher pneumolysin levels and more pronounced cell damage.

In conclusion, CCL20 was shown to be a crucial chemotactic chemokine during acute pneumococcal meningitis, attracting neutrophils to the subarachnoid space. The neutrophil response in pneumococcal meningitis that is guided to the subarachnoid space through the chemokine/receptor pair CCL20/CCR6 is important for limiting bacterial outgrowth.
